# Supplementary material for: HiFi chromosome-scale diploid assemblies of the grape rootstocks 110R, Kober 5BB, and 101–14 Mgt
Source: Sci Data. 2022 Oct 28;9:660. doi: 10.1038/s41597-022-01753-0 (PMC9616894; doi:10.1038/s41597-022-01753-0)
Supplement: Supplementary file 1 — Supplemental figure 3 [file 41597_2022_1753_MOESM1_ESM.pdf]

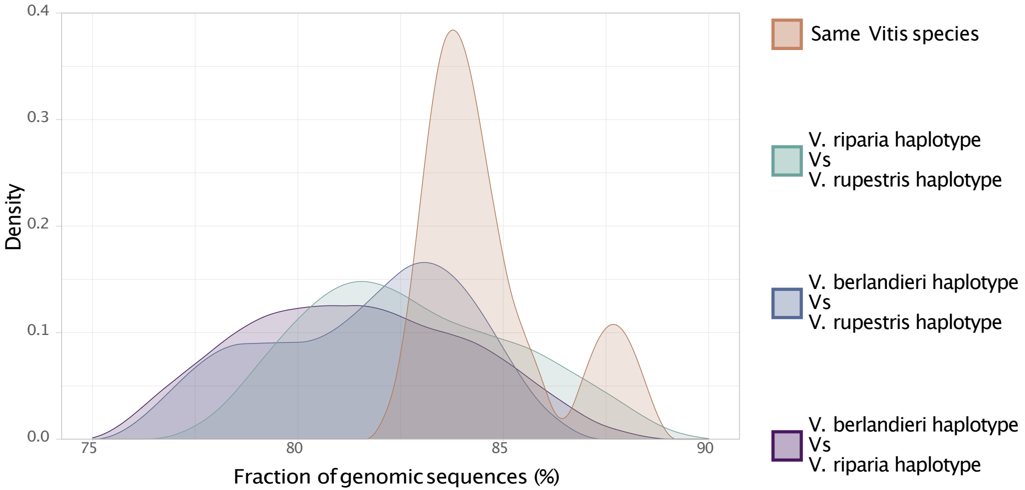

Supplemental figure 3: Colinearity between haplotypes Fraction of the genomic sequences in colinear block of genes between each pair of haplotypes. In average, 82.4% ±2.6% of the genomic sequences are comprised inside colinear blocks, with very little differences between haplotypes assigned to same the Vitis species or to distinct ones. Density plot was produced with adjust = 1 , n=4096, kernel ="cosine" parameters.
