# Supplementary material for: HiFi chromosome-scale diploid assemblies of the grape rootstocks 110R, Kober 5BB, and 101–14 Mgt
Source: Sci Data. 2022 Oct 28;9:660. doi: 10.1038/s41597-022-01753-0 (PMC9616894; doi:10.1038/s41597-022-01753-0)
Supplement: Supplementary file 3 — Supplemental figure 1 [file 41597_2022_1753_MOESM3_ESM.pdf]

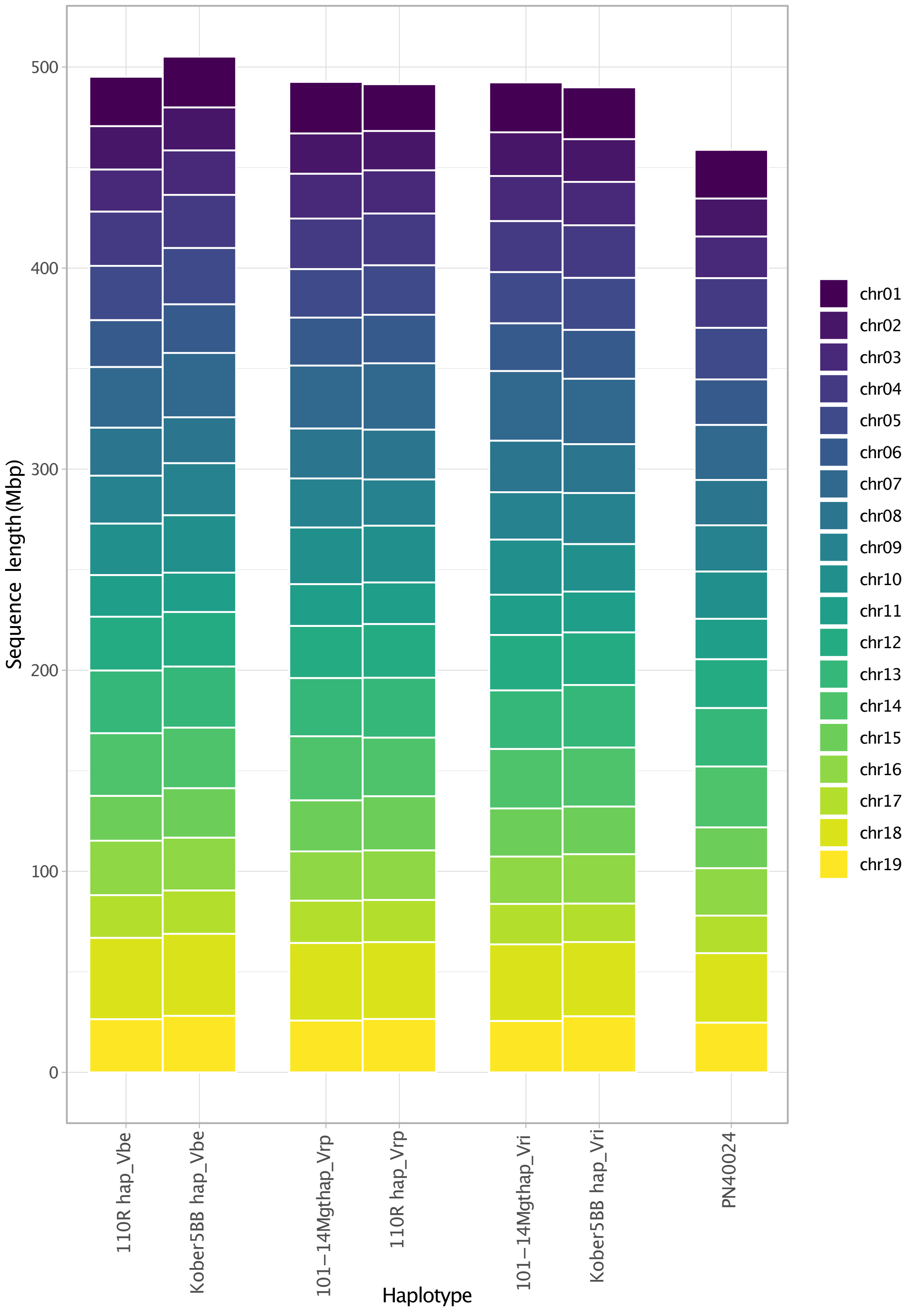

Supplemental figure 1: Reconstructed pseudomolecules size comparison. Size of the reconstructed pseudomolecules for each haplotype of the three rootstocks and V. vinifera PN40024 genome.
