# Supplementary material for: HiFi chromosome-scale diploid assemblies of the grape rootstocks 110R, Kober 5BB, and 101–14 Mgt
Source: Sci Data. 2022 Oct 28;9:660. doi: 10.1038/s41597-022-01753-0 (PMC9616894; doi:10.1038/s41597-022-01753-0)
Supplement: Supplementary file 4 — Supplemental figure 2 [file 41597_2022_1753_MOESM4_ESM.pdf]

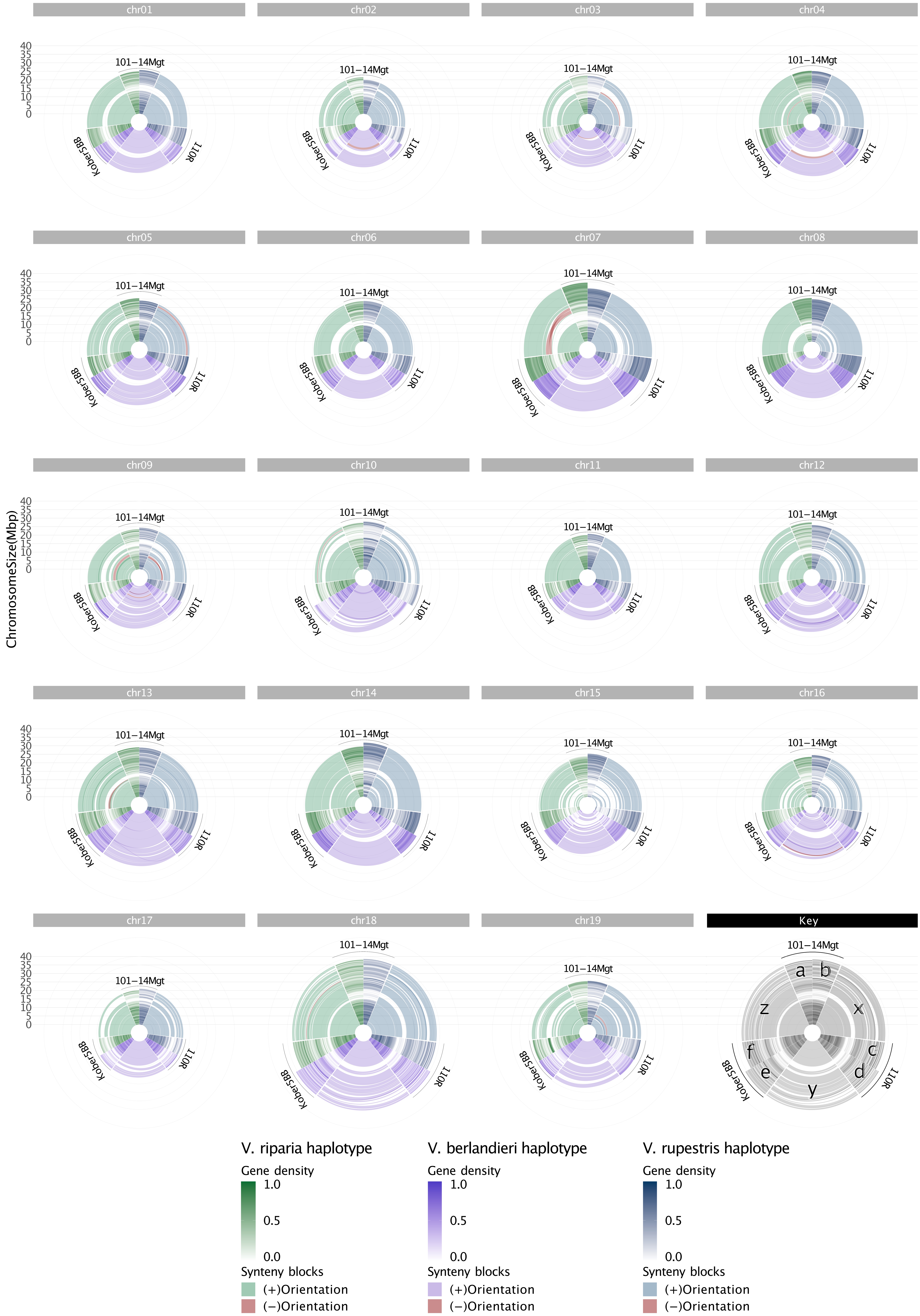

Supplemental figure 2: Three-waysynteny between 101−14Mgt,110R, and Kober 5BB rootstocks highlights the colinearity between siblings. Gene density along 101−14Mgt haplotypes inherited from V. riparia (a) and V. rupestris (b), 110R haplotypes from V. rupestris (c) and V. berlandieri (d), Kober 5BB haplotypes from V. berlandieri (e) and V. riparia (f). Syntenic regions between the V. rupestris haplotypes of 101−14Mgt and Kober 5BB (x), the V. berlandieri haplotypes of 110R and Kober 5BB (y), and the V. riparia haplotypes of 101−14Mgt and 110R (z).
